# An Investigation of the Clinical Utility of the Proposed ICD-11 and DSM-5 Diagnostic Schemes for Eating Disorders Characterized by Recurrent Binge Eating in People with a High BMI

**DOI:** 10.3390/nu10111751

**Published:** 2018-11-13

**Authors:** Marly Amorim Palavras, Phillipa Hay, Angélica Claudino

**Affiliations:** 1 Eating Disorders Program (PROATA), Department of Psychiatry, Universidade Federal de São Paulo (UNIFESP), São Paulo 04017030, Brazil; marlypalavras@gmail.com; 2 Translational Health Research Institute, School of Medicine, Western Sydney University, Sydney 2751, Australia; p.hay@westernsydney.edu.au

**Keywords:** Bulimia Nervosa, binge-eating disorder, Diagnostic and Statistical Manual of Mental Disorders, International Classification of Diseases

## Abstract

The aims of this paper were to compare (1) the proportion of participants diagnosed with threshold or subthreshold Bulimia Nervosa (BN) and Binge Eating Disorder (BED) (clinical utility), and (2) the severity of participants’ clinical features and mental Health-Related Quality of Life (HRQoL) (convergent validity), when diagnosed according to either the Diagnostic and Statistical Manual of Mental Disorders—5th edition (DSM-5) or the proposed International Classification of Diseases 11th edition (ICD-11) schemes. One hundred and seven adult men and women, with a high Body Mass Index (BMI) were evaluated by interview to confirm their eating disorder diagnoses. All participants completed self-report assessments of current symptoms and mental HRQoL. The majority of participants in either diagnostic scheme were included in the main categories of BN or BED (102/107, 95% in the ICD-11 and 85/107, 79% in the DSM-5). Fewer individuals received a subthreshold other or unspecified diagnosis with the ICD-11 compared to the DSM-5 scheme (5% vs. 21%). No significant differences in demographic, clinical features or mental HRQoL of participants with complete or partial BN or BED were found between diagnostic categories. Compared to the DSM-5, the proposed ICD-11 was not over inclusive, i.e., it did not appear to include people with less severe and potentially less clinically relevant symptoms. These results support the greater clinical utility of the ICD-11 whilst both schemes showed convergent validity.

## 1. Introduction

Currently two diagnostic classification systems guide the field of mental disorders, the Diagnostic and Statistical Manual of Mental Disorders in its 5th edition (DSM-5) [1] and the International Classification of Diseases, now in its 10th edition (ICD-10) [2]. The 11th edition of the ICD- is in preparation [3,4]. A World Psychiatric Association Global Survey (WPA-WHO) involving 4887 psychiatrists in 44 countries indicated that 79.2% of the sample often or almost always/always used one of these formal classification systems as part of their day-to-day clinical work [5]. The DSM-5, which covers mental health exclusively, is very commonly used in the United States, United Kingdom, Australasia, and in international research. The ICD-10 is more extensive, as it includes all medical diagnoses, and it is the official government classification scheme in the majority of countries worldwide [2,5]. Further, the two schemes differ substantively in their structure, definitions and diagnostic guidelines, particularly concerning disorders characterized by recurrent binge eating such as Binge Eating Disorder (BED) and Bulimia Nervosa (BN) [6,7].

In both schemes, BED is now included as the third major eating disorder after BN and anorexia nervosa (AN). Both systems also became more inclusive aiming to reduce the formerly very high number of cases (around 60%) diagnosed within the residual poorly specified categories. DSM-5 now has two such categories—Other Specified Feeding or Eating Disorder (OSFED) and Unspecified Feeding or Eating Disorder (UFED), respectively. OSFED has five diagnostic groups: (1) atypical anorexia nervosa; (2) BN of low frequency and/or limited duration; (3) BED of low frequency and/or duration; (4) purging disorder; and (5) night eating syndrome. UFED is the residual category with no specific criteria [1]. The proposed ICD-11 guidelines [8,9] have such category—Other Feeding or Eating Disorder (OFED). The proposed OFED is closer to the DSM-5 UFED, in being a more general, nonspecific category of people who fall outside diagnostic criteria for a main eating disorder. The main features of OFED are the inclusion of atypical eating behaviors; symptoms that do not fulfill criteria for other Feeding and Eating Disorder (FED), for another mental and behavioral disorder, for another health condition, or that are not secondary to the use of substance or medication interfering on the central nervous system; symptoms and behaviors that are not sanctioned by cultural aspects; and finally, symptoms that impact on the person’s personal, social, or work life.

In the DSM-5 [1] and the provisional ICD-11 guidelines [9], the essential features of binge eating are: (1) frequency of once weekly or more; (2) loss of control over the binge eating episode; and (3) marked distress. ICD-11 differs in the inclusion of subjective binge eating episodes (SBEs), where the amount consumed is normal or small although subjectively considered as large by the individual. This is supported by a large body of research [10,11,12,13]. A second point of difference between the DSM-5 and proposed ICD-11 criteria for BN and BED is the duration of binge eating episodes. In the DSM-5 this is at least 3 months for both disorders. In the proposed ICD-11 the recommendation is at least one month for BN and three months for BED except where the binge eating episodes are very frequent and the diagnosis of BED may be made for shorter period, such as one month. Third, the presence of diagnostic specifiers are required as diagnostic criteria for BED in DSM-5, while these are not essential in the provisional ICD-11.

In the DSM-5, a mental disorder is defined as “associated with significant distress or disability in social, occupational, or other important activities” (p. 20, [1]) and the proposed ICD-11 states the same [2]. Whilst functional disability is not further specified for either BN or BED, many studies do attest to the poor mental Health-Related Quality of Life (HRQoL) and role impairment, defined as “days in which the person was unable to perform the usual work or other activities, e.g., caring for children in the home” [14]. As the ICD-11 proposes broader symptomatic criteria, it is important to know if these define a syndrome of individuals with less impairment or poorer HRQoL than would be found using the DSM-5 scheme, and who may not meet the over-arching requirement for social or functional impairment as suggested in recent epidemiological studies [15,16].

Thus, the aims of this study were to compare (1) the proportion of participants diagnosed with threshold or subthreshold BN and BED (clinical utility); and (2) the severity of clinical features and mental HRQoL of these categories (convergent validity), in either scheme. We hypothesized that the broader criteria proposed in the ICD-11 would decrease the number of diagnoses of unspecified or not otherwise specified categories to a greater degree than in the DSM-5.

## 2. Materials and Methods

### 2.1. Procedures

Data were collected in a sample of 107 individuals of both genders, with threshold and subthreshold BN or BED, who attended the second phase of assessment for eligibility to participate in a randomized controlled trial (RCT) testing the efficacy of a new psychological intervention to treat overweight or obese people with BN or BED [17]. Participants were recruited from the waiting list of a Brazilian university center specialized in the treatment of eating disorders and advertisements in printed, internet and oral media. Assessment started with a brief screening interview by telephone. Volunteers were then assessed in person with the Mini International Neuropsychiatric Interview (MINI) [18,19], administered by psychiatrists, and height and weight measured with calibrated scales and a stadiometer.

**Figure 1:**
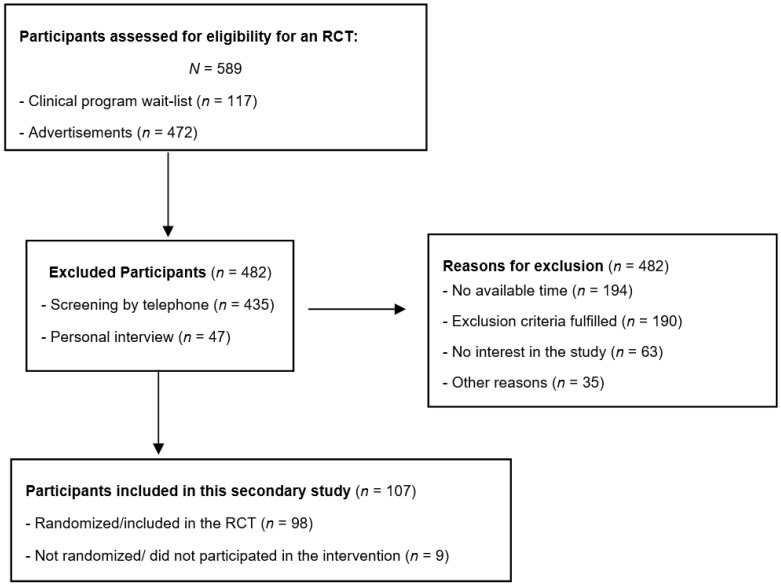
Flow chart of patient selection.

A third assessment phase involved the administration of a semi-structured interview named the Eating Disorder Examination Edition 17.0 D (EDE) [20]. This interview was used both to confirm the eating disorder diagnoses of participants and to collect detailed data on overall eating disorder symptoms and behaviors. One author (MAP), who had been trained at the Centre for Research on Eating Disorders at Oxford, administered the EDE and made the diagnoses of each patient based on DSM-5 criteria [1] and the provisional ICD-11 diagnostic guidelines [8,9]. For the purpose of this secondary study, inclusion criteria were: participants that met DSM-5 diagnostic criteria for BN, BED, OSFED–BN, OSFED-BED, or UFED, were aged ≥18 years and had a Body Mass Index (BMI) between 27 and 39.9 kg/m^2^. Thus, in this secondary study, 107 participants were selected, including 98 people who enrolled in the RCT and 9 participants who participated in all selection and recruitment assessments, but declined to enter the treatment intervention. Figure 1 shows the participant flow and reasons for exclusion during recruitment phases.

### 2.2. Measures

The assessment measures used for the study are detailed below:

1. Mini International Neuropsychiatric Interview (MINI) [18,19]: this structured interview is reliable and validated for diagnoses according to the DSM-5 [1]. The MINI version 5 [21] assessing DSM-IV diagnoses [22] has been translated into Portuguese. Thus, the MINI-5 was used in this study, with modifications to coding according to DSM-5 criteria, as in the MINI-7 [19].
2. Eating Disorder Examination (EDE) Edition 17.0D [20]: the EDE is a semi-structured interview. It assesses eating disorder features. It has four subscales measuring levels of dietary restraint, eating, shape and weight concerns. The global score is a mean of subscale scores. In this study, the new Version 17.0D item scoring, that included “being in control” as a reason for the behavior or cognition, was used. EDE version 16.0 [23] has been translated to Brazilian/Portuguese by researchers from the Universidade Federal do Rio de Janeiro (Silvia Freitas, José Carlos Appolinario), by authors of this paper (MAP, AC) and by an eating disorder specialist and member of the Eating Disorder Program (Christina Morgan—CM). A certified translator then back translated it into English. An author of the EDE (O’Connor, M) approved the final version. The translated EDE interrater reliability and concurrent validity were tested and found to be satisfactory. In the validity study, the diagnoses were made according to the eating disorder module of the SCID-I/P interview [24] (Portuguese version) [25]. Diagnostic agreement between the Portuguese EDE version 16.0 and the SCID I/P (both testing DSM-IV diagnoses) was found to be moderate (Kappa = 0.66; *n* = 149), with a Cronbach *α* of 0.91 (95% CI: 0.88 0.92) (unpublished data provided by AC). For the purpose of the present study, small adjustments were made by MAP, so that the Portuguese version used was consistent with the 17th version of the EDE [20], in order to derive DSM-5 categories. Cronbach *α* in this sample was 0.66, *n* = 107.
3. Binge Eating Scale (BES) [26]: the BES is a 16-item self-report instrument that was translated and validated in a Brazilian sample [27]. The BES measures frequency and severity of binge eating. The Brazilian version has undergone psychometric assessment and has a cut-off point for normality of 17 for the screening of eating disorders in obese individuals seeking treatment for weight loss. Cronbach *α* in this sample was 0.83, *n* = 106.
4. Loss of Control over Eating Scale (LOCES) [28]. This 24-item self-report scale measures loss of control during binge eating episodes in the last 4 weeks, with a mean score of 1.70 (SD = 5.72). It has been translated into Brazilian/Portuguese. This translated version has adequate psychometric properties (factor analysis and convergent validity) [29]. Cronbach *α* in this sample was 0.91, *n* = 107.
5. Depression, Anxiety and Stress Scale (DASS-21) [30,31] was used in its translated and validated Brazilian/Portuguese version [32]. The DASS is a 21-item self-report instrument that measures the presence and severity of depressive symptoms, anxiety and stress with a cut-off point for normality of 9 for depression, 7 for anxiety and 14 for stress. Cronbach *α* in this sample was 0.92, and for each subscale was: stress 0.85, depression 0.89, and anxiety 0.77, *n* = 107.
6. Health-related quality of life was assessed with the 12-item Short Form Health Survey (SF-12) [33]: a self-report questionnaire. It has two scales, the Physical Health Component Summary scale (PCS) and Mental Health Component Summary scale (MCS). The English language version has sound psychometric properties and it has been translated into Brazilian/Portuguese [34]. Cronbach *α* in this sample was 0.79, *n* = 106.
7. Disability was asked with a question closely similar to the ‘days out of role’ questions employed in the Australian National Survey of Mental Health and Well-Being [35]. Participants were asked to write for the past 4 weeks a response to, *“on how many days, if any, were you unable to complete your work, study or household responsibilities because of any problem with your (physical or emotional) health”?* An author (MAP) translated this text.

### 2.3. Statistical Analyses

Frequency and percentage statistics were used to report the distribution of diagnoses including OSFED, UFED and OFED categories for each scheme. Chi square statistic (χ^2^) and Fisher’s Exact Test were used to compare the frequency distributions of BN, BED, and OSFED/UFED or OFED for each scheme. The t-test or the Mann-Whitney U test were employed for parametric or non-parametric data and/or very small group sizes respectively, with correction of *p* to < 0.01 for multiple testing. SPSS v.23 was used for analyses [36].

### 2.4. Ethics

The RCT study was approved by the Human Research Ethics Committee of the Universidade Federal de São Paulo (UNIFESP), Brazil (CAAE 43874315.4.0000.5505). All participants provided written informed consent. Access to the data was limited to investigators and authorized researchers. The trial was formally registered in the U.S. National Institutes of Health Clinical, trial registration number NCT02464345, on 1 June 2015.

## 3. Results

One hundred and seven participants were included in this study. The majority of the participants were women (96%), and white (75%). A large minority were married (44%), and completed an under-graduate level of education (43%). The mean age was 40.07 years (SD 11.67), and mean BMI was 33.69 (SD 3.32). The sample included 50 (47%) participants with twice weekly Objective Binge Eating Episodes (OBEs) and 36 (34%) with weekly OBEs in the past 3 months. The remainder had a mix of OBEs and SBEs with the exception of one participant who had only SBEs.

Diagnostic evaluation using DSM-5 criteria resulted in 15/107 (14%) participants with BN, 70/107 (65.4%) with BED, 8/107 (7.5%) with OSFED-BN, 5/107 (4.7%) with OSFED-BED, and 9/107 (8.4%) participants with UFED. Applying the proposed ICD-11 criteria, there were 20/107 (18.7%) with BN, 82/107 (76.6%) with BED and 5/107 (4.7%) participants with OFED. The provisional ICD-11 diagnostic guidelines included 102/107 participants (95%) in the main categories of BN or BED compared to DSM-5 with 85/107 (79%), and 5/107 (5%) versus 22/107 (21%) with other or unspecified diagnosis (χ^2^ = 20.265, df = 1, *p* ˂ 0.001, Fisher’s Exact Test).

Comparative clinical features of the participants diagnosed according to either scheme are reported in Table 1. There were no significant differences in eating disorder or other psychiatric symptom severity, HRQoL, days out of role or BMI between participants with BN, BED or OSFED/UFED/OFED diagnosed according to DSM-5 or the proposed ICD-11.

## 4. Discussion

This study compared the clinical utility and convergent validity of the two main classification systems for psychiatric disorders—the DSM-5 [1] and the provisional ICD-11 [8,9]. Threshold and subthreshold diagnoses of BN and BED were derived using the “gold standard” semi-structured interview for eating disorders—the EDE-17.0D [20]. In both systems, a majority of participants met full BN or BED criteria. This supports the improved clinical utility of both schemes over the former DSM-IV [22] and ICD-10 [2] where a larger proportion of people did not meet full criteria [6,37].

In the present study, a greater number of participants received a main diagnosis of BN or BED with the provisional ICD-11 scheme compared to the DSM-5. Despite the inclusion of some less severe cases in those diagnosed according to ICD-11, overall there were no significant differences in levels of eating disorder or general psychopathology, BMI, mental or physical HRQoL, or role impairment between diagnoses defined by either classificatory system. These data do not support the findings reported by Mitchison et al. [16] where there was less impairment in people with less severe recurrent binge eating. A possible explanation is that the Mitchison study was based on the results of a community sample and this study used a clinical sample of people with high BMIs and an eating disorder who were seeking treatment, i.e., people potentially with more severe illness. Indeed, the present study included a higher proportion of people with twice-weekly OBEs, 58.1% (50/86) compared to 40.5% (158/390) in the Mitchison sample; and in the latter study those with twice weekly binge eating had poorer mental HRQoL, whether associated or not with marked distress.

Thus, the results of this study demonstrated that regardless of the classificatory system used, significant levels of associated general psychopathology and impact on mental HRQoL were observed in people with disorders characterized by recurrent binge eating. Our findings support those of Hudson’s et al. [38], in which a high association between lifetime comorbid psychiatric disorders and role impairment in people with BED and BN was reported.

Different approaches guide these two classificatory systems, in particular the fact that the proposed ICD-11 diagnostic guidelines do not provide a “check-list” for diagnostic criteria. The diagnosis is based on a description of the essential features of the conditions, i.e., symptoms and characteristics that clinicians expect to find in all cases of the disorder. Reed [37] highlights that a large number of people with mental disorders around the globe may not receive treatment as a consequence of diagnoses being restricted by over rigid criteria. We anticipate that clinicians will likely find the ICD-11 scheme more user friendly, as it allows for greater clinician judgement [3] and is more flexible than the DSM-5. More of their patients may thereby receive definitive diagnosis and also, be eligible for care that is contingent on having a main diagnosis. On the other hand, the strict criteria of the DSM-5 are more applicable to the research setting where there is a need for tight criteria for reproducibility of studies.

A strength of this study was the use of an interview considered the gold standard (the EDE) for providing eating disorders diagnoses. Limitations include the small numbers of participants with ICD-11 BN and OSFED/UFED/OFED, with a subsequent risk of TYPE II error; a sample characterized by individuals who are overweight or obese; no participants with AN or other eating disorders; and the moderate alpha level of the EDE. Further, the BES has been validated in people with obesity and this sample included a small number (13.1%) with BMI 27.5–29.9. A final limitation is that the present study reports on a secondary analysis of data from a RCT testing the efficacy of psychological interventions for overweight people with bulimic disorders and used an EDE version, which does not collect the distress criterion across both OBEs and SBEs. However, the vast majority, 77 of 82 participants, had distress associated with OBEs.

## 5. Conclusions

The present article found that both the DSM-5 and ICD-11 diagnostic schemes had a low proportion of participants with a poorly specified (OSFED/UFED) diagnosis. Further, those included as BN or BED in the ICD-11 were not less severe than the ones with BN or BED in the DSM-5 groupings. The increased flexibility of the proposed ICD-11 may extend the clinical utility of this scheme compared to the DSM-5. However, further research is needed in larger samples of people with BN, people who are not overweight, and in people with other eating disorders diagnoses.

## Figures and Tables

**Table 1:** Comparative clinical features of the study participants (*N* = 107).

Clinical Features	Bulimia Nervosa			Binge Eating Disorder			^#^ Osfed/Ufed/Ofed			All
	DSM–5 (*n* = 15)	ICD–11 (*n* = 20)		DSM–5 (*n* = 70)	ICD–11 (*n* = 82)		DSM–5 (*n* = 22)	ICD–11 (*n* = 5)		*N* = 107
	Median (IQR)		Z (*p*) ^†^	Median (IQR)		Z (*p*)	Median (IQR)		MWU Z (*p*)	
EDE global	3.59 (3.27–3.83)	3.80 (2.35–3.83)	−0.044 (0.965)	2.48 (1.95–2.88)	2.27 (1.93–2.41)	−1.233 (0.217)	2.23 (1.98–2.74)	2.60 (2.01–2.77)	−0.196 (0.845)	2.57 (2.03–3.23)
EDE restriction	3.00 (2.70–3.60)	2.20 (1.00–3.80)	−0.394 (0.694)	1.60 (0.08–2.40)	1.40 (0.50–2.20)	−0.994 (0.320)	1.60 (0.60–2.20)	1.40 (1.00–1.60)	−0.157 (0.875)	1.80 (0.80–2.40)
EDE eating concern	2.00 (1.90–2.70)	3.00 (2.60–3.20)	−1.228 (0.220)	1.20 (0.60–2.20)	0.90 (0.20–1.40)	−1.605 (0.108)	1.20 (0.20–2.40)	0.60 (0.40–2.40)	−0.197 (0.844)	1.40 (0.60–2.40)
EDE shape concern	5.00 (4.25–5.37)	4.62 (2.62–4.75)	−0.961 (0.336)	3.62 (2.62–4.50)	3.44 (3.06–4.20)	−0.230 (0.818)	3.50 (3.00–4.62)	3.62 (2.88–3.88)	−0.118 (0.906)	3.75 (2.87–4.62)
EDE weight concern	4.20 (3.30–4.70)	3.20 (3.20–3.80)	−1.488 (0.137)	3.20 (2.40–4.20)	3.20 (2.70–3.67)	−0.263 (0.793)	3.20 (2.80–3.75)	2.80 (2.60–3.60)	−0.472 (0.637)	3.20 (2.60–4.20)
Binge Eating Scale	35.00 (30.50–41.00)	32.00 (31.00–33.00)	−0.963 (0.335)	29.00 (22.00–33.00)	28.00 (21.00–32.50)	−0.677 (0.498)	28.00 (24.00–33.00)	25.00 (25.00–25.00)	−1.061 (0.289)	30.00 (23.00–35.00)
LOCES	3.67 (3.29–4.18)	3.50 (3.08–3.62)	−0.917 (0.359)	3.21 (2.79–3.50)	3.12 (2.62–3.43)	−0.564 (0.573)	3.33 (2.70–3.54)	3.45 (3.41–3.50)	−0.746 (0.456)	3.33 (2.83–3.58)
DASS depression	22.00 (16.50–33.00)	24.00 (24.00–28.00)	−0.044 (0.965)	12.00 (6.00–20.00)	14.00 (7.00–17.00)	−0.066 (0.948)	16.00 (10.00–24.00)	10.00 (8.00–12.00)	−1.178 (0.239)	14.00 (8.00–24.00)
DASS anxiety	12.00 (5.00–14.00)	22.00 (4.00–26.00)	−0.615 (0.539)	8.00 (4.00–14.00)	17.00 (2.00–20.00)	−0.896 (0.370)	18.00 (2.00–22.00)	18.00 (6.00–18.00)	−0.277 (0.782)	10.00 (4.00–16.00)
DASS stress	28.00 (20.00–34.00)	30.00 (26.00–32.00)	−0.307 (0.799)	18.00 (12.00–28.00)	21.00 (14.00–27.00)	−0.059 (0.953)	26.00 (16.00–28.00)	20.00 (18.00–22.00)	−0.511 (0.609)	20.00 (14.00–28.00)
SFMCS	30.12 (26.63–38.70)	34.35 (34.32–40.81)	−1.702 (0.089)	36.34 (26.60–42.70)	37.14 (31.24–42.94)	−0.499 (0.618)	36.70 (32.61–42.74)	42.03 (35.74–44.78)	−1.136 (0.256)	36.02 (27.42–42.71)
SFPCS	46.27 (43.51–52.98)	47.00 (46.50–55.90)	−0.567 (0.570)	50.24 (41.97–56.36)	48.77 (40.64–56.60)	−0.273 (0.785)	47.00 (41.14–55.90)	50.39 (47.13–53.58)	−0.431 (0.667)	49.82 (41.52–56.20)
Body Mass Index (kg/m^2^)	32.60 (29.72–34.31)	32.70 (31.30–34.60)	−0.655 (0.513)	33.60 (31.12–35.85)	34.65 (32.63–37.02)	−0.964 (0.335)	33.80 (32.37–36.90)	33.89 (30.70–35.29)	−0.823 (0.411)	33.64 (31.08–35.90)
Days out of role/28 days	10.00 (1.00–15.00)	4.00 (2.00–10.00)	−0.482 (0.630)	5.00 (2.00–12.00)	1.00 (0–11.00)	−1.341 (0.180)	2.00 (0–10.00)	0 (0–0)	−1.466 (0.143)	5.00 (0–12.00)

^#^ Note ICD-11 has OFED as the only subthreshold category. ^†^ MWU Z = Mann Whitney U Z statistic. BED = binge eating disorder; BN = bulimia nervosa; DASS = Depression, Anxiety and Stress Scale; DSM-5 = Diagnostic and Statistical Manual of Mental Disorders 5th ed; EDE = Eating Disorder Examination; ICD-11 = International Classification of Diseases 11th ed.; IQR = Interquartile Range; LOCES = Loss of Control Over Eating Scale; OFED = Other Feeding or Eating Disorder; OSFED = Other Specified Feeding or Eating Disorder; SFMCS = Short Form Health Survey Mental Health Component Summary score; SFPCS = Short Form Health Survey Physical Health Component Summary score; UFED = Unspecified Feeding or Eating Disorder.

## Trial Registration

U.S. National Institutes of Health—clinical trial registration number NTC02464345. URL: clinicaltrials.gov/ct2/show/NCT0246434.
